# Maladies tropicales et pauvreté : impact sur les droits de la femme et de l'enfant - journée scientifique de la Sfmtsi DU 25 Mai 2022

**DOI:** 10.48327/mtsi.V2I2.2022.245

**Published:** 2022-06-28

**Authors:** Jean JANNIN, Jacques CHANDENIER, Jean DELMONT, Alain EPELBOIN, Françoise GAY-ANDRIEU, Pierre GAZIN, Catherine GOUJON, Pierre MARTY, Claire TANTET

**Affiliations:** SFMTSI. Hôpital Pitié-Salpêtrière - Pavillon Laveran, 47-83 Boulevard de l'Hôpital, 75651 Paris cedex 13

**Keywords:** Droits, Femme, Enfant, Pauvreté, Trachome, Addiction, Opioïdes, Tramadol, Leishmaniose cutanée, Épilepsies, Albinisme oculocutané, Saturnisme, Maladies tropicales négligées, Nigeria, Sénégal, Afrique subsaharienne, Rights, Women, Children, Poverty, Trachoma, Addiction, Opioids Tramadol, Cutaneous leishmaniasis, Epilepsy, Oculocutaneous albinism, Lead poisoning, Neglected tropical diseases, Nigeria, Senegal, Sub-Saharan Africa

Cette Journée scientifique de printemps de la SFMTSI propose de dresser un panorama des déterminants des maladies de la pauvreté et des conséquences dramatiques qu'elles entraînent sur les droits de la femme et de l'enfant.

Le cycle des maladies tropicales, souvent favorisées par la pauvreté et généralement évitables, s'inscrit dans un cercle vicieux où la pauvreté, renforcée par de nombreuses calamités humaines ou naturelles, favorise lourdement l'apparition de pathologies qui à leur tour se répercutent sur les droits de l'homme, en particulier ceux de la femme et de l'enfant, populations particulièrement vulnérables.

Rappelons que cette problématique fondamentale du droit à la santé, appliquée en particulier aux femmes et aux enfants, fut une préoccupation majeure dans la réflexion ayant abouti à l’élaboration du concept de maladies tropicales négligées (MTN) par l'OMS, qui fut menée en collaboration avec le comité des Nations unies sur les droits économiques, sociaux et culturels, par le rapporteur spécial sur le droit au meilleur état de santé.

En effet, outre les souffrances physiques et psychologiques qu'elles causent, ces maladies font payer dans ce contexte un lourd tribut économique aux communautés touchées, en raison notamment de la perte de productivité. Leurs conséquences, symptômes et séquelles contribuent à leur tour au cercle vicieux de la pauvreté, de la maladie, de la stigmatisation et de la discrimination dont sont victimes les populations souvent les plus défavorisées de la planète. Les graves séquelles obèrent les capacités de développement des enfants et compromettent leur accès à une vie meilleure.

