# Supplementary material for: Tropical diseases and poverty: impact on women's and children's rights - scientific day of the Sfmtsi, 25 may 2022
Source: Med Trop Sante Int. 2022 Jun 28;2(2):mtsi.V2I2.2022.245. [Article in French] doi: 10.48327/mtsi.V2I2.2022.245 (PMC9326779; doi:10.48327/mtsi.V2I2.2022.245)
Supplement: Related texts

# Research article: Impact des maladies tropicales et de la pauvreté sur les droits de la femme et de l'enfant

**Published:** 2022-06-28

**Authors:** Jean JANNIN

**Affiliations:** SFMTSI. Hôpital Pitié-Salpêtrière - Pavillon Laveran, 47-83 Boulevard de l'Hôpital, 75651 Paris cedex 13

**Figure 1:** 

Le cercle vicieux de la pauvreté et de l'impact de nombreux déterminants et facteurs aggravants sur les maladies tropicales conduit à ce que de nombreuses populations paient un prix exorbitant en termes de répercussions de ces affections sur ces inégalités, accentuant encore la pauvreté et réduisant plus encore leurs chances futures.

L'analyse des déterminants de la santé selon le modèle de convergence de Smolinski (2003) met en évidence le large spectre des facteurs impactant négativement ces populations ainsi que leur coût humain et social.

De plus, il convient de noter que les femmes et les enfants sont les principales victimes de ce cercle vicieux. Pis encore, outre les répercussions et séquelles physiques ou les conséquences économiques et sociales, la structure philosophique de nos sociétés fait que l'application de traditions, croyances ou pratiques socio-culturelles particulières entraîne des atteintes majeures aux droits humains en général et très spécifiquement aux droits de la femme et de l'enfant.

Si la Déclaration des droits de l'homme de 1789 a plus de deux siècles, elle ne prenait en compte que les droits civils et politiques et elle ignorait les femmes, les enfants et les esclaves. Celle de 1948 palliera en partie au moins à ces manques et introduira le droit à la santé. Rappelons pour mémoire que les principes de non-discrimination, d'accès aux soins, d'accessibilité, de qualité et d'universalité définis dès 1946 par l'OMS pour une approche de la santé basée sur les droits de l'homme sont bien trop souvent passés par pertes et profits.

# Research article: Impact des droits des femmes sur la santé en milieu tropical

**Published:** 2022-06-28

**Authors:** Claire TANTET

**Affiliations:** Hôpital Avicenne, Bobigny Hôpital Bichat, Paris

Cette présentation a pour objectif d'ouvrir le débat sur la santé des femmes dans le monde et particulièrement en milieu tropical. En effet, leur santé est impactée par les inégalités de genre. Leur accès au soin dépend de nombreux facteurs notamment socio-économiques, culturels et religieux, mais aussi de leur travail et de leur mode de vie au sein de la famille. Ceci est vrai de façon universelle et présente des spécificités en milieu tropical. Nous explorons dans cette présentation certaines violences faites aux femmes ayant un impact direct sur leur santé, certaines pathologies propres aux femmes ainsi que le droit universel des femmes. Enfin nous ferons le lien entre maladies tropicales négligées et santé des femmes. Puis nous ouvrirons la discussion pour réfléchir aux possibilités de changement et d’évolution.

# Research article: Maladies tropicales et pauvreté : impact sur les droits de l'enfant

**Published:** 2022-06-28

**Authors:** Johanna THOMSON, Anja GAO

**Affiliations:** Médecins sans frontières (MSF)

Résumé non communiqué

# Research article: Les maladies cécitantes (trachome) et leur impact sur les droits de la femme et de l'enfant

**Published:** 2022-06-28

**Authors:** Serge RESNIKOFF

**Affiliations:** University of New South Wales, Sidney

Véritable marqueur de la pauvreté, le trachome est une maladie oculaire due à *Chlamydia trachomatis* qui affecte les populations les plus défavorisées d'Afrique, d'Amérique latine, d'Asie et d'Océanie. Même si le nombre de personnes vivant dans les communautés à risque diminue progressivement, le trachome reste un problème de santé publique dans une soixantaine de pays. Bien qu'il débute peu après la naissance, le trachome n'entraîne des complications invalidantes qu'après des années d’évolution, généralement à l’âge adulte. Ces complications sont d'autant plus sévères que le nombre de réinfections au cours de la vie a été plus élevé. À cet égard les femmes sont particulièrement à risque car ce sont elles qui sont en contact avec les enfants, principal réservoir de *C. trachomatis.* Ce sont donc essentiellement elles qui deviennent aveugles. Incurables, les déficiences visuelles dues au trachome ont un impact économique et social important. Non seulement elles entraînent une perte de productivité mais surtout elles nécessitent un soutien permanent pour l'accomplissement des actes essentiels de la vie des personnes concernées. C'est généralement aux jeunes enfants qu'incombe le rôle d'assistant de vie et de guide d'aveugle, ce qui les prive de fréquenter l’école.

L’élimination du trachome en tant que problème de santé publique représente donc non seulement un moyen de lutter contre une maladie particulièrement invalidante, mais aussi de contribuer à la réduction de la pauvreté et de l'exclusion scolaire. Malheureusement, après une période de succès dans cette entreprise, l'heure n'est plus à l'euphorie car les moyens deviennent de plus en plus limités tandis que les problèmes d'insécurité, de persistance et de recrudescence ont tendance à augmenter.

# Research article: Profils et trajectoires des femmes usagères de drogues au Sénégal : les consommatrices de tramadol, une « catégorie à part »

**Published:** 2022-06-28

**Authors:** Rose André FAYE

**Affiliations:** TransVIHMI-IRD/CRCF-CNHU, Dakar, Sénégal

En 2011, les femmes usagères de drogues injectables identifiées par l'enquête UDSEN étaient moins nombreuses que les hommes (69/507) et davantage touchées par le VIH. En 2021, elles sont toujours minoritaires parmi les patients suivis au Centre de prise en charge intégrée des addictions de Dakar (CEPIAD), premier centre de soins pour les usagers de drogue (UD) de l'Afrique de l'Ouest offrant la méthadone. Depuis 2016, ce modèle pilote de soins est évalué. L'objectif de cette présentation est de décrire les profils des femmes UD au Sénégal et d'analyser les particularités liées à l'usage de tramadol en comparaison avec d'autres pays d'Afrique. Cette présentation est issue d'une thèse sur les trajectoires des femmes UD, associée à deux projets de recherche

(CODISEN/CODISOCS) et d'une revue de littérature. L'approche qualitative associe des observations et des récits de vies collectés entre 2017 et 2021 auprès de 34 femmes. Nos données montrent quatre profils de femmes utilisant des drogues injectables en produit principal (cocaïne, héroïne, tramadol) : des jeunes femmes du monde de la nuit et du divertissement, des jeunes femmes des quartiers populaires et des associations de travailleuses du sexe (TS), des femmes connues dans le milieu des UD comme « anciennes junkies », et des femmes dépendantes aux médicaments isolés. Ces dernières constituent une « catégorie à part », socialement insérées. Elles sont entrées dans l'addiction pour avoir abusé du tramadol qui leur a été prescrit comme antalgique. La littérature internationale montre que la consommation des médicaments opioïdes est notamment liée au taux de prescription des médecins et aux modèles de consommation des femmes. En outre, les régions d'Afrique de l'Ouest et du Centre font face à une crise liée au tramadol. Des saisies importantes témoignent d'un usage non médical du tramadol en Afrique de l'Ouest (ONUDC 2019). Les données disponibles au Burkina Faso, au Cameroun, au Niger et au Mali indiquent que plusieurs catégories sont représentées parmi les personnes usagères de tramadol : femmes, prostitués, conducteurs de motos taxis, cultivateurs… Ces derniers ont souvent recours à un usage détourné du tramadol pour mieux travailler et supporter l’épuisement. Les récits de vie montrent une diversité de profils qui suggère de multiplier les réponses en fonction des catégories. L'analyse des parcours montre que certaines (mais pas toutes) cumulent plusieurs vulnérabilités : TS, personnes vivant avec le VIH, UD. Il est nécessaire d'approfondir la question de l'entrée dans l'addiction par les prescriptions médicales et des usages du tramadol en Afrique.

# Research article: La leishmaniose cutanée chez la femme et l'enfant : entre stigmatisation et atteinte des droits humains

**Published:** 2022-06-28

**Authors:** Mourad MOKNI

**Affiliations:** Chef du service de Dermatologie, hôpital La Rabta, Tunisie

La leishmaniose, une maladie tropicale négligée, a des liens forts mais complexes avec la pauvreté. La morbidité de la leishmaniose affecte de manière disproportionnée les plus pauvres de la population mondiale. Dans les zones endémiques, l'augmentation du risque d'infection est due aux mauvaises conditions de logement et d'assainissement, à l'absence de mesures de protection individuelle et aux facteurs migratoires qui amènent des hôtes non immunisés en contact avec les phlébotomes infectés. La pauvreté est associée à une mauvaise nutrition et à d'autres maladies infectieuses, ce qui augmente le risque qu'une personne une fois infectée évolue vers une maladie cliniquement plus sévère. Le manque d'accès aux soins de santé entraîne des retards dans le diagnostic et dans le traitement approprié et accentue la morbidité et la mortalité liées à la leishmaniose, en particulier chez les femmes et les enfants. Les femmes sont doublement désavantagées, et rencontrent des obstacles plus élevés aux soins de santé et une stigmatisation plus intense que les hommes. Elles risquent de se voir interdire de toucher leurs enfants pour éviter de les infecter, d’être abandonnées par leurs maris, de se cacher et ne pas accéder aux soins et d’être stigmatisées suite aux séquelles cicatricielles de la maladie. Toutes ces contraintes affectent directement leurs enfants. La morbidité globale exprimée en DALY (espérance de vie corrigée de l'incapacité, EVCI) est estimée à 0,58 par 100 000 habitants. Cependant, cette estimation est controversée car elle considère uniquement les lésions actives de leishmaniose cutanée et néglige les séquelles cicatricielles défigurantes. Certains auteurs suggèrent de multiplier par 10 l'estimation de la morbidité de la maladie tenant compte des séquelles de la maladie. Cette présentation est une revue de la leishmaniose cutanée comme maladie liée à la pauvreté, impactant davantage les franges sociales les plus fragiles, et plus particulièrement les femmes et les enfants. Elle discute également la grande stigmatisation liée aux séquelles cicatricielles, ainsi que l'atteinte globale à la qualité de vie et aux droits humains des patientes et de leurs enfants.

# Research article: Épilepsies tropicales et séquelles neurologiques

**Published:** 2022-06-28

**Authors:** Jérémy JOST

**Affiliations:** Institut d’Épidémiologie et de Neurologie Tropicale, université de Limoges

Dans la majorité des pays de la zone tropicale, l’épilepsie reste un problème de santé publique majeur. Parmi les 70 millions de personnes avec une épilepsie dans le monde, 80 % vivent en zone tropicale où le déficit thérapeutique (personnes avec une épilepsie ne bénéficiant pas du traitement dont elles ont besoin) est estimé supérieur à 75 % (et très souvent proche de 100 % en milieu rural). Dans de nombreux pays, le manque de ressources médicales et de médicaments, l'absence de formation des personnels de santé, les idées fausses et les préjugés qui entourent la maladie constituent autant d'obstacles à l'accès aux soins. La prévalence de l’épilepsie présente de fortes variations dans la zone intertropicale, s'expliquant principalement par des causes spécifiques. Le point commun entre toutes ces zones est généralement un déficit thérapeutique élevé et des contextes socio-culturels conduisant à une stigmatisation importante. Depuis 2010, après une large campagne de sensibilisation mondiale sur un déficit de traitement supérieur à 75 %, les recherches interventionnelles et/ou les initiatives se sont multipliées à la recherche d'un modèle de soin efficient adapté à ces pays aux revenus faibles et intermédiaires, afin de bénéficier au plus grand nombre. En 2019, l'Organisation mondiale de la santé (OMS), la Ligue internationale contre l’épilepsie (ILAE) et le Bureau international pour l’épilepsie (IBE) ont publié conjointement un rapport intitulé « Épilepsie : un impératif de santé publique ». Les thématiques mises en avant afin de sensibiliser (à nouveau) la communauté internationale sont le principe de fardeau (sanitaire, psychologique et psychiatrique, social comme économique), le stigma et la discrimination (en parlant notamment de violation des droits de l'homme), le déficit de traitement (toujours supérieur à 70%), la prévention (au moins 25 % des cas seraient évitables) et la nécessité de voir émerger des politiques de santé publique intégrant l’épilepsie.

# Research article: L'albinisme oculocutané en Afrique subsaharienne a-t-il sa place parmi les maladies tropicales négligées ?

**Published:** 2022-06-28

**Authors:** Robert AQUARON

Bien qu’étant une affection génétique, l'albinisme oculocutané (AOC) spécialement en Afrique subsaharienne peut être considéré comme une MTN car les albinos ou « personnes atteintes d'albinisme » (PAA) font très souvent l'objet de discriminations économiques, sociales, culturelles, morales, médicales et physiques. La naissance d'un enfant albinos de deux parents de peau noire a toujours été une énigme pour les populations africaines. Considéré comme un authentique « Africain blanc », l'albinos est ressenti comme appartenant à un monde magique qui fait de lui une créature humaine intermédiaire et inachevée, mi-homme, mi-Dieu, doué de pouvoirs innés qui peut faire le bien ou le mal.

Cette particularité a fait des albinos la proie de crimes rituels et de mutilations en vue d'utiliser certains de leurs organes, bras, jambes, organes génitaux pour la préparation de talismans ou gris-gris qui sont supposés porter chance, santé, prospérité, et conjurer les maladies ou les mauvais sorts. Toutes ces exactions étaient seulement connues localement et n’étaient pas diffusées dans les médias étatiques ou internationaux. En 2008, des crimes rituels à grande échelle en Tanzanie et au Burundi ont été rapportés dans la presse écrite et parlée africaine et internationale par une ONG canadienne, Under The Same Sun (UTSS). Son président, Peter Ash, a porté ce tragique événement à la connaissance du président de la République de Tanzanie qui a condamné vigoureusement ces assassinats et le commerce des parties de leurs corps. Il a continué son action en faveur des albinos en sollicitant les instances internationales, l'ONU, en particulier l'OMS et l'UNESCO.

Son action a été déterminante pour l'adoption par l'assemblée générale des Nations unies le 13 juin 2013 d'une résolution au sujet des agressions et des discriminations à l'encontre des albinos. En 2014, l'organisation a proclamé le 13 juin Journée internationale de sensibilisation à l'albinisme. Cette date du 13 juin a été fêtée officiellement avec brio en 2016 par l'organisation d'une journée au siège de l'UNESCO à Paris comportant 4 sessions : un panel de médecins spécialistes de l'albinisme, un film sur le traitement chirurgical des cancers de la peau en Tanzanie, une exposition photos « blancébène » et un panel d'associations d'albinos sur « l'albinisme au quotidien, témoignages ». Au niveau national et international, afin de combattre toutes les atteintes contre les droits fondamentaux des personnes avec albinisme, se sont développées des associations dont les buts étaient de réunir les albinos et leur famille pour leur apporter les informations nécessaires sur la cause de leur affection, et d'informer les parents sur la façon d’élever ces enfants si fragiles. En Afrique subsaharienne, la première association a été créée au Mali en 1993 par le chanteur Salif Keita, puis au Cameroun en 1996. Actuellement, elles existent dans la plupart des pays francophones et anglophones.

# Research article: A perfect storm in Nigeria – drought, gold, and Sharia law result in childhood deaths due to lead poisoning

**Published:** 2022-06-28

**Authors:** Magrit VON BRAUN

**Affiliations:** TerraGraphics International Foundation (TIFO), 121 S Jackson St, Moscow, ID 83843, États-Unis

A perfect storm of environmental, cultural and economic conditions caused an unprecedented epidemic of lead poisoning in Nigeria in 2010. Due to the high price of gold, subsistence farming families, including women and children, engaged in artisanal mining to triple family income. However, prevailing Sharia practices required that the ore be brought into the home for crushing and grinding. Unaware that the ore was more that 10% lead, severe soil contamination resulted in mass poisoning of more than 30,000 people and the deaths of 400-500 children. Lead poisoning added to endemic disease challenges, including malnutrition, cholera, malaria and polio. Since 2010, Médecins sans frontières (MSF), TerraGraphics International Foundation (TIFO), Nigerian civil and traditional governments, and other international organizations have collaborated in implementing a cleanup, medical treatment, and public health intervention program to decrease childhood mortality rates from >30% to <2%. The cleanup involved more than 350 villagers, 80 local government personnel, and 28 international advisors. More than 2,000 children were provided chelation therapy treatment. Daunting cultural, logistic, socio-economic challenges were encountered in adapting sophisticated engineering and medical protocols to remote locations in ten villages with little resources or infrastructure. A permanent, coordinated Institutional Controls Program that includes medical, environmental, safer mining, community engagement and health promotion components has been developed by state, local, federal government officials, with support from international partners. These programs are essential for as long as subsistence artisanal gold mining is important to the economy. The response to the lead poisoning crisis in Nigeria demonstrates that modest assistance can effectively respond to these challenges.

**Figure:** Journée scientifique de la SFMTSI (ex SPE) - Hommage à Alain CHIPPAUX (1928 - 2022)
